# Sodium, potassium and blood pressure in Australian schoolchildren: exploring differences by sex and weight status—a cross-sectional study

**DOI:** 10.1038/s41440-025-02489-1

**Published:** 2026-01-18

**Authors:** Carley A. Grimes, Karen Lim, Lachlan Clark, Mark Woodward, Ewa A. Szymlek-Gay, Miaobing Zheng, Caryl A. Nowson, Kristy A. Bolton

**Affiliations:** 1 Institute for Physical Activity and Nutrition, School of Exercise and Nutrition Sciences, Deakin University, Geelong, VIC Australia; 2 School of Exercise and Nutrition Sciences, Deakin University, Geelong, VIC Australia; 3 The George Institute for Global Health, University of New South Wales, Sydney, NSW Australia; 4 The George Institute for Global Health, School of Public Health, Imperial College London, London, UK; 5 School of Health Sciences, Faculty of Health & Medicine, University of New South Wales, Sydney, NSW Australia

**Keywords:** Sodium, Potassium, Blood pressure, Paediatric, Hypertension

## Abstract

Dietary sodium and potassium intake play a key role in the regulation of blood pressure (BP). This study investigated whether 24- urinary sodium, potassium and sodium-to-potassium ratio were associated with blood pressure in Australian schoolchildren aged 4–12 years, and if the association between 24-h urinary sodium and blood pressure was moderated by body weight. Twenty-four-hour urine, blood pressure, and anthropometry were collected from 755 schoolchildren (mean age 9.3 (SD 1.8) years). Multiple linear regression with adjustment for covariates was conducted. The mean sodium excretion was 2419 (SD 1052) mg/d. Seventeen percent of children had elevated blood pressure. There were no overall associations between 24-h sodium or potassium excretion and blood pressure in adjusted regression models. However, in adjusted regression analysis stratified by sex, there was a positive association between 24-h urinary sodium and systolic blood pressure z-score among girls (b-coefficient 0.10 [95% CI 0.03, 0.18], *p*value = 0.01, *n* = 342). No other sex differences were observed. Body weight significantly moderated the association between sodium excretion and SBP (p for interaction = 0.002). In children living with obesity, sodium excretion was positively associated with systolic blood pressure z-score (b-coefficient 0.75 [95% CI 0.00, 1.51], *p*value = 0.05, *n* = 21). In conclusion, sodium excretion in this sample exceeded recommended levels for healthy development and almost a fifth of children had elevated blood pressure. For optimal health across life, public health interventions aiming to reduce the elevated cardiovascular risk of raised blood pressure in children are likely to be most effective by reducing sodium intake in conjunction with promoting healthy weight.

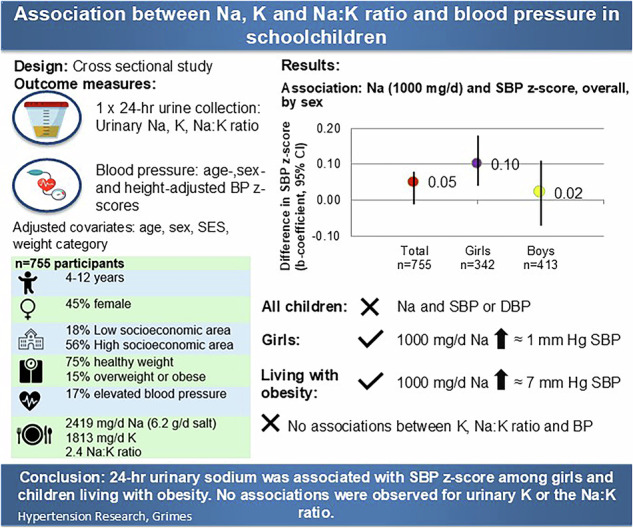

## Introduction

Targeting early life predictors of blood pressure, such as sodium intake, are important to promote healthy blood pressure trajectories across the life course. In adults, the importance of a diet low in sodium and high in potassium to reduce blood pressure [1–3] and the risk of cardiovascular disease [1, 4, 5] is well known. In children, dietary sodium and potassium have also been shown to influence blood pressure. In population-based cross-sectional studies of children, higher intakes of sodium have been associated with raised systolic blood pressure (SBP) [6, 7] and meta-analyses of intervention trials conducted in children have shown that reductions in dietary sodium lead to modest reductions in blood pressure [8, 9]. Furthermore, there is some evidence to suggest that the effects of sodium intake on blood pressure may be more pronounced in children with excess body weight [9–12]. Evidence related to potassium intake and blood pressure during childhood is less consistent. A meta-analysis, of three intervention trials and one cohort study, found no association between potassium intake and blood pressure in children [2]. However, more recent longitudinal studies have reported that higher intakes of potassium during childhood predict small reductions in SBP during adolescence and early adulthood [13, 14]. There is also some evidence to suggest that a lower ratio of sodium-to-potassium during childhood is protective of raised blood pressure levels [13, 15]. It is well established that blood pressure tracks over the life course [16], with raised levels during childhood increasing the risk of high blood pressure later in life [17]. Elevated blood pressure across childhood has also been shown to contribute to early vascular and target organ damage [18] as well as cardiovascular endpoints in midlife [19]. Therefore, understanding the relationship between dietary intake of sodium and potassium on blood pressure in early life is important.

Twenty-four-hour (24-h) urinary sodium is considered the most accurate method to assess dietary sodium intake [20]. This method captures all sodium consumed in the diet, including from food sources and discretionary salt added during cooking or at the table [20]. It is also not subject to errors associated with dietary recall methods (e.g. misreporting of foods and beverages) or limited by the completeness of sodium information available in food composition databases [20]. Among the paediatric population, relatively few studies have used 24-h urine collections to assess the relationship of sodium and potassium intakes with blood pressure [21, 22]. The present study seeks to extend knowledge in this area by assessing the cross-sectional association of 24-h urinary sodium, potassium, and the sodium-to- potassium ratio with blood pressure in Australian schoolchildren aged 4–12 years. A secondary aim was to determine if the association between 24-h urinary sodium and blood pressure was moderated by body weight.

## Methods

### Study design and subjects

**Fig. 1:**
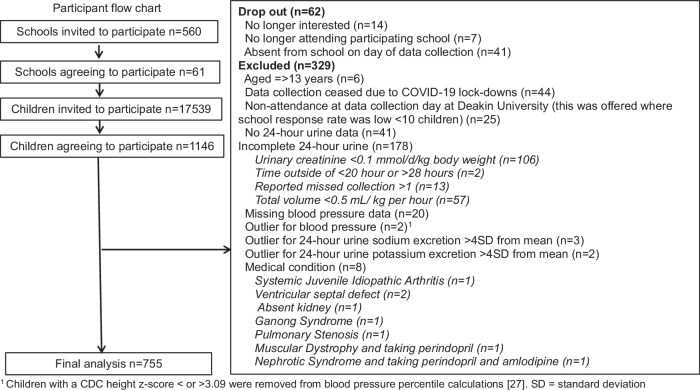
Participant flow chart. ^1^ Children with a CDC height z-score < or >3.09 were removed from blood pressure percentile calculations [33]. SD standard deviation

This was a cross-sectional study, with data collected in independent samples of children aged 4–12 years attending primary schools located in Victoria, Australia in 2010–13 (Cohort 1) and 2018–19 (Cohort 2). Outcomes related to sodium and potassium intakes and the food sources of these nutrients from Cohort 1 were previously reported [23]. The study was repeated in 2018–19 (Cohort 2) to assess if sodium intake was reduced in children living in Victoria, Australia, following the implementation of a 4-year salt reduction intervention (2015–2019) [24]. To maximise participant numbers with 24-h urine collections and blood pressure measurements, the current study combines data from both cohorts. Data collection procedures were the same at both time-points. Government and non-government schools located in Victoria were identified via an online school locator. In 2010–13, a convenience sample of schools was selected, and the school principal was invited to participate. In 2018–19, in the first instance the previously participating 2010–13 schools were approached; following exhaustion of this list schools were recruited that were comparable to those selected in 2010–13 with regards to (i) the mix of non-government vs. government schools and (ii) the spread of schools across levels of socioeconomic disadvantage, based on school postcode and corresponding Socio-Economic Indexes for Areas (SEIFA) [25]. In total, 61 schools agreed to participate (school response rate 11%) (Fig. 1). All children aged 4–12 years were eligible to participate. In Victoria, Australia children aged 4, turning 5 by the 30th of April in the year they start school are eligible to commence elementary school. Of the 17, 539 children invited to participate, 1146 (7%) agreed. Of the consenting participants, 291 were excluded and 62 dropped out of the study, leaving a sample of 793 in the analyses (Fig. 1). This included 647 from Cohort 1 data collection period and 146 from Cohort 2. Ethical approval was granted by Deakin University Ethics Committee (ID numbers: EC62-2009 and HEAG-H 01_2018). Permission to conduct research in government schools was provided by the Victorian Department of Education and Training (ID numbers: 2011_001151 and 2018_003666). Written consent to participate was obtained from school principals and parents; assent was obtained from children.

### Data collection

#### Demographic and anthropometric data

The primary carer of each participant completed a questionnaire collecting child and parent demographic characteristics, which included date of birth (child and parent), sex (child and parent), existing health conditions (child), medication use (child), birth weight, and educational attainment (parent) [24, 26]. Each child was classified according to national thirds of socio-economic status depending on the postcode of their school using the Australian SEIFA index (Socio-Economic Indexes for Areas, Index of Relative Socio-Economic Disadvantage) [25]. The child’s weight and height were measured following standard protocols [26]. Age and sex-adjusted BMI z-scores were calculated [27]. Children were grouped into weight categories (underweight, healthy weight, overweight, obese) [28].

#### 24-hour urine collection

Children completed a 24-h urine sample on either a school day or non-school day (weekend, public holiday or school holiday) [26]. School day collections commenced at the start of school, whereas non-school day collections commenced at any time suitable to the participant and parents recorded start and finish times on collection materials. Written instructions, tailored for either a school or non-school day collection, were provided to parents, and simplified pictorial instructions were provided for children. The collection commenced by discarding the first urine; following this, all urine was collected for a 24-h period in containers provided. For school day collections research staff were available throughout the day to monitor and co-ordinate collection materials so children could collect their urine. At the end of the day, children were given their collection materials and instructions to continue the collection at home. During the collection period completed at home, any missed collections or spillages were recorded by parents on a urine collection slip and returned to research staff. Returned samples were transported to a commercial pathology laboratory (Dorevitch Pathology) where total volume was recorded and analysis of sodium, potassium and creatinine completed. Urinary sodium and potassium concentrations were assessed using indirect ion selective electrodes and urinary creatinine concentration was assessed using the Jaffe reaction on the Siemens Advia 2400 analyser (Siemens Healthcare). The CV for sodium and potassium was <1% and for creatinine it was 3.25%. For analysis, urinary measures were standardised to a 24-h collection period i.e., (24 h/reported urine duration (hours)) x urinary measure. Existing criteria were used to determine if samples were incomplete: urinary creatinine excretion was <0.1 mmol/kg body weight/day [29], collection time was <20 h or >28 h [30], >1 collection was reported as missing [30], total volume was <0.5 mL/kg per hour [30, 31]. Samples (*n* = 178) meeting ≥1 of these criteria were excluded (Fig. 1).

#### Blood pressure

Blood pressure was measured in seated children, using the right arm and an appropriate sized cuff, after 10 min of rest [26]. A digital automatic blood pressure machine (OMRON HEM-907) was used, and three measurements were completed at 1-minute intervals. The mean of the final two measures was used in analysis. Age-, sex- and height-adjusted percentiles of blood pressure were calculated using the 2017 American Academy of Paediatrics (AAP) Guidelines [32] and corresponding US population reference data [33]. Children with a height z-score < or >3.09 were excluded [33]. Blood pressure percentiles were converted to z-scores using the inverse normal distribution. Participants were categorised as having normal blood pressure (systolic blood pressure [SBP] and diastolic blood pressure [DBP] <90th percentile), or elevated blood pressure (SBP or DBP ≥90th percentile) [32].

### Data analysis

Analyses were conducted using STATA SE (version 17, StataCorp LP, College Station, TX). Data were reported with descriptive statistics using mean and 95% CI for continuous variables and number, percentage (%) and 95% CI for categorical variables. A *P* value of <0.05 was considered significant. Sodium to salt conversions were based on the molecular weights of sodium (23 g/mol) and sodium chloride (58.5 g/mol). The percentage of children exceeding the age-specific upper level for sodium intake (i.e., 2000 mg/d for 4–8 years and 2300 mg/d for 9–13 years) [34] was calculated. As no differences in urinary electrolytes was found at the data collection time points, the two data sets were combined for analysis. Multiple linear regression models were used to assess the associations between independent variables: (i) sodium excretion (1000 mg/d), (ii) potassium excretion (1000 mg/d), (iii) sodium-to-potassium molar ratio, and systolic and diastolic blood pressure z-scores (dependent variables). Four regression models were fitted; model 1: unadjusted; model 2: adjusted for age, sex, socioeconomic disadvantage, weight category and day of urine collection (school vs. non-school day); and model 3: model 2 plus potassium intake when sodium was the independent variable, or vice versa, model 2 plus sodium intake when potassium intake was the independent variable; model 4: model 2 plus birth weight (g). Birth weight was included as a covariate due to its inverse association with blood pressure later in life [35]. Model 4 was completed in a reduced sample size (*n* = 605) as *n* = 149 children had missing birth weight data, and 1 child was excluded as their recorded birth weight was deemed implausible (297 g). Interactions between all urinary electrolytes (sodium, potassium, and sodium-to-potassium ratio) and sex were examined and stratified findings presented. To assess if the association between salt intake and blood pressure differed depending on weight category (underweight/healthy weight, overweight, and obese) interaction terms between urinary sodium and weight category were added to the regression model and stratified findings presented. All interaction analyses were based on model 2. Due to the relatively low number of children grouped within the obese category, we completed a sensitivity analysis where children were grouped into two weight categories (e.g., underweight/healthy weight and overweight/obese). Regression diagnostics were used to check model assumptions, this included variance inflation factors for multicollinearity, visual assessment of normality of residual distributions and linearity using a component-plus-residual plot. All regression models used robust standard errors to account for clustering of students within schools.

## Results

The mean age of children was 9 years, just under half of the sample were girls and 63% of children had a parent with a university/tertiary qualification. Seventy five percent of children were of a healthy weight. Eighty three percent of children were classified as having normal blood pressure, and 17% with elevated blood pressure. Mean 24-h urinary sodium excretion was 105 mmol/d (equivalent to 6.2 g/d of salt) and 74% of children exceeded the daily upper level for sodium intake. Mean 24-h urinary potassium excretion was 46 mmol/d (1813 mg/d) and mean molar sodium-to-potassium ratio was 2.4 (Table 1).

**Table 1:** Demographic, health, and urinary electrolyte data of children aged 4–12 years participating in the Salt and Other Nutrient Intakes in Children study, overall and stratified by sex

Characteristic	All (*n* = 755)	Girls (*n *= 342, 45%)	Boys (*n* = 413, 55%)	*P* value^a^
	n or mean (% or SD)	n or mean (% or SD)	n or mean (% or SD)	
Age (years)	9.3 (1.8)	9.3 (1.7)	9.3 (1.9)	0.80
**Socioeconomic disadvantage of school**^**b**^				
Low (e.g., most disadvantaged)	139 (18%)	72 (21%)	67 (16%)	0.15
Middle	190 (25%)	93 (27%)	97 (23%)	
High (e.g,. least disadvantaged)	426 (56%)	177 (52%)	249 (60%)	
**Parental educational attainment**^**c**^				
Low	139 (22%)	61 (21%)	78 (22%)	0.83
Mid	104 (16%)	44 (15%)	60 (17%)	
High	412 (63%)	188 (64%)	224 (62%)	
**School type**				
Government	512 (68%)	223 (65%)	289 (70%)	0.38
Non-government	243 (32%)	119 (35%)	124 (30%)	
**Blood pressure (BP)**				
SBP (mm Hg)	102 (10)	102 (11)	103 (10)	0.14
DBP (mm Hg)	60 (9)	61 (9)	59 (9)	0.02
**2017 AAP BP criteria**^**d**^				
SBP percentile	57 (28)	55 (29)	58 (26)	0.27
DBP percentile	49 (25)	51 (25)	46 (24)	0.002
SBP z-score	0.22 (0.95)	0.18 (1.01)	0.25 (0.90)	0.35
DBP z-score	-0.001 (0.80)	0.07 (0.81)	-0.09 (0.80)	0.003
Normal BP (SBP and DBP <90th percentile)	624 (83%)	285 (83%)	339 (82%)	0.68
Elevated BP (SBP or DBP ≥90th percentile)	131 (17%)	57 (17%)	74 (18%)	
**Weight category**^**e**^				
Underweight	74 (10%)	35 (10%)	39 (9%)	0.62
Healthy weight	569 (75%)	252 (74%)	317 (77%)	
Overweight	191 (12%)	43 (13%)	48 (12%)	
Obese	21 (3%)	12 (3%)	9 (2%)	
BMI z-score	0.08 (1.03)	0.10 (1.02)	0.06 (1.04)	0.61
Birth weight (g)^f^	3389 (645)	3301 (593)	3459 (676)	0.005
**24-hour urinary electrolytes**				
Sodium (mmol)	105 (46)	98 (42)	111 (48)	0.001
Sodium (mg)	2419 (1052)	2248 (963)	2561 (1101)	0.001
Salt equivalent (g)	6.2 (2.7)	5.7 (2.4)	6.5 (2.8)	0.001
Potassium (mmol)	46 (17)	45 (16)	48 (18)	0.02
Potassium (mg)	1813 (676)	1743 (618)	1871 (717)	0.02
Sodium: potassium molar ratio	2.4 (1.1)	2.3 (1.0)	2.5 (1.1)	0.06
**Day of urine collection**				
School day	331 (44%)	146 (43%)	185 (45%)	0.56
Non-school day	424 (56%)	196 (57%)	228 (55%)	

*BP* blood pressure, *SBP* systolic blood pressure, *DBP* diastolic blood pressure, *AAP* American Academy of Paediatrics

^a^*P* value determined via Pearson’s χ^2^ test or independent *t* test

^b^Based on school postcode and corresponding national index of relative socio-economic disadvantage [1]

^c^*n *= 655 (missing data *n* = 100). Participants were grouped as either low: includes those with some or no level of high school education, mid: includes those with a technical/trade certificate or high: includes those with a university/tertiary qualification.

^d^BP percentiles, z-scores, and categories based on 2017 American Academy of Paediatrics Guidelines [2]

^e^Weight classification based on the International Obesity Task Force BMI reference cut-offs [3]

^f^*n* = 605 due to missing data *n* = 149 and *n* = 1 excluded as reported birth weight deemed implausible (297 g)

### 24-hour urinary electrolyte excretion and blood pressure among all children

In the overall sample, in the unadjusted model there was a positive association between both 24-h urinary sodium and potassium excretion and SBP z-score; however, these become non-significant after adjustment (Table 2). There was no indication of an association between sodium or potassium excretion and DBP z-score, nor of an effect of the sodium-to-potassium ratio on SBP or DBP z-score (Table 2).

**Table 2:** Association between 24-h urinary sodium (1000 mg/d), potassium (1000 mg/d) excretion and the sodium-to-potassium molar ratio with blood pressure z-scores among children aged 4–12 years participating in the Salt and Other Nutrient Intakes in Children study (*n* = 755)

24-h urinary electrolyte and model fitted	SBP z-score	DBP z-score
	b-coefficient (95% CI), *P* value	b-coefficient (95% CI), *P* value
	R^2^, *P* value	R^2^, *P* value
**Sodium (1000 mg/d)**		
Model 1	**0.08 (0.03, 0.12), 0.003** R^2^ = 0.007, 0.003	–0.02 (–0.06, 0.02), 0.32 R^2^ = 0.0007, 0.32
Model 2	0.05 (–0.01, 0.11), 0.08 R^2^ = 0.02, 0.06	–0.02 (–0.08, 0.03), 0.38 R^2^ = 0.02, <0.001
Model 3	0.03 (–0.02, 0.09), 0.21 R^2^ = 0.02, 0.07	–0.01 (–0.07, 0.05), 0.66 R^2^ = 0.02, <0.001
Model 4^a^	0.05 (–0.01, 0.12), 0.11 R^2^ = 0.03, 0.01	–0.02 (–0.07, 0.04), 0.66 R^2^ = 0.04, <0.001
**Potassium (1000 mg/d)**		
Model 1	**0.13 (0.04, 0.22), 0.006** R^2^ = 0.009, 0.006	–0.05 (–0.13, 0.04), 0.29 R^2^ = 0.002, 0.29
Model 2	0.08 (–0.01, 0.18), 0.08 R^2^ = 0.02, 0.07	–0.06 (–0.14, 0.03), 0.20 R^2^ = 0.02, <0.001
Model 3	0.06 (–0.03, 0.16), 0.18 R^2^ = 0.02, 0.07	–0.05 (–0.14, 0.04), 0.29 R^2^ = 0.02, <0.001
Model 4^a^	0.10 (–0.002, 0.21), 0.05 R^2^ = 0.04, 0.02	–0.01 (–0.10, 0.07), 0.73 R^2^ = 0.03, <0.001
**Sodium to potassium ratio**		
Model 1	–0.02 (–0.06, 0.3), 0.45 R^2^ = 0.0004, 0.45	–0.01 (–0.06, 0.03), 0.50 R^2^ = 0.0004, 0.50
Model 2	–0.02 (–0.07, 0.03), 0.42 R^2^ = 0.02, 0.12	–0.01 (–0.06, 0.03), 0.56 R^2^ = 0.02, 0.001
Model 4^a^	–0.01 (–0.07, 0.05), 0.68 R^2^ = 0.03, 0.02	–0.01 (–0.07, 0.04) R^2^ = 0.04, <0.001

Model 1: unadjusted

Model 2: age, sex, socioeconomic disadvantage, weight category, day of urine collection

Model 3: model 2 + potassium where sodium is independent variable + sodium where potassium is independent variable

Model 4: Model 2 + birth weight (kg).

Bold values indicate statistical significance *P* value < 0.05

^a^*n* = 605 due to missing data for birth weight *n *= 149 and *n *= 1 excluded as reported birth weight deemed implausible (297 g)

### 24-hour urinary electrolyte excretion and blood pressure by sex

When stratified by sex, adjusting for age, socioeconomic disadvantage, weight category and day of urine collection, there was a significant positive association between 24-h urinary sodium (1000 mg/d; equivalent to 2.5 g salt) and SBP z-score among girls (b-coefficient 0.10 [95% CI 0.03, 0.18], *p*value = 0.01, n = 342) but not in boys (b-coefficient 0.02 [95% CI –0.07, 0.10], *p*value = 0.69, n = 413) (Table 3, Model 2). Additional adjustment for potassium excretion attenuated the association between urinary sodium and SBP z-score in girls (b-coefficient 0.06 [95% CI –0.02, 0.14], *p*value = 0.14, (*n* = 365). In the reduced sample with birth weight data, the positive association between sodium excretion and SBP z-score remained among girls after adjustment with birth weight (*n* = 239) (Table 3, Model 4). However, observed sex differences were not statistically significant (interaction terms *p*value ≥ 0.15 for all comparisons using Model 2). Among boys (*n* = 413), a positive association between potassium excretion and SBP z-score was observed in the unadjusted model; however, this association was no longer significant after adjustment for covariates (Table 3). No other sex-specific differences were found for potassium excretion or the sodium-to-potassium ratio in relation to SBP or DBP z-score.

**Table 3:** Association between 24-h urinary sodium (1000 mg/d), potassium (1000 mg/d), and sodium-to-potassium molar ratio with systolic and diastolic blood pressure z-scores among children aged 4–12 years participating in the Salt and Other Nutrient Intakes in Children study, stratified by sex

24-h urinary electrolyte	SBP z-scores		DBP z-scores	
	Girls (*n* = 342)	Boys (*n* = 413)	Girls (*n* = 342)	Boys (*n* = 413)
	b-coefficient (95% CI), *P* value	b-coefficient (95% CI), *P* value	b-coefficient (95% CI), *P* value	b-coefficient (95% CI), *P* value
	R^2^, *P* value	R^2^, *P* value	R^2^, *P* value	R^2^, *P* value
**Sodium (1000 mg/d)**				
Model 1	**0.11 (0.04, 0.17), 0.001** R^2^ = 0.01, 0.002	0.05 (–0.03, 0.13), 0.19 R^2^ = 0.004, 0.19	–0.04 (–0.10, 0.02), 0.22 R^2^ = 0.002, 0.21	0.01 (–0.06, 0.07), 0.81 R^2^ = 0.0001, 0.81
Model 2	**0.10 (0.03, 0.18), 0.008** R^2^ = 0.03, 0.03	0.02 (–0.07, 0.11), 0.69 R^2^ = 0.03, 0.14	–0.05 (–0.13, 0.03), 0.19 R^2^ = 0.01, 0.49	–0.01 (–0.08, 0.07), 0.87 R^2^ = 0.01, 0.87
Model 3	0.06 (–0.02, 0.14), 0.14 R^2^ = 0.02, 0.01	–0.02 (–0.10, 0.07), 0.71 R^2^ = 0.03, 0.08	-0.06 (-0.14, 0.03), 0.17 R^2^ = 0.04, 0.06	–0.01 (–0.09, 0.08), 0.89 R^2^ = 0.02, 0.02
Model 4^a^	**0.10 (0.002, 0.20), 0.04** R^2^ = 0.04, 0.14	-0.03 (-0.07, 0.13), 0.57 R^2^ = 0.04, 0.02	–0.04 (–0.12, 0.05), 0.39 R^2^ = 0.01, 0.51	0.002 (–0.07, 0.07), 0.94 R^2^ = 0.03, 0.49
**Potassium (1000 mg/d)**				
Model 1	0.15 (–0.02, 0.32), 0.09 R^2^ = 0.009, 0.09	**0.11 (0.01, 0.22), 0.03** R^2^ = 0.01, 0.03	–0.06 (–0.19, 0.07), 0.35 R^2^ = 0.002, 0.35	–0.02 (–0.12, 0.08), 0.70 R^2^ = 0.003, 0.70
Model 2	0.12 (–0.08, 0.32), 0.23 R^2^ = 0.02, 0.29	0.06 (–0.07, 0.18), 0.36 R^2^ = 0.03, 0.12	–0.06 (–0.18, 0.06), 0.31 R^2^ = 0.01, 0.34	–0.05 (–0.17, 0.07), 0.39 R^2^ = 0.02, 0.12
Model 3	0.06 (–0.15, 0.27), 0.56 R^2^ = 0.02, 0.01	0.05 (–0.09, 0.19), 0.51 R^2^ = 0.03, 0.08	–0.08 (–0.21, 0.06), 0.24 R^2^ = 0.04, 0.06	–0.07 (–0.21, 0.06), 0.28 R^2^ = 0.02, 0.02
Model 4^a^	0.19 (–0.02, 0.39), 0.07 R^2^ = 0.04, 0.20	0.06 (–0.08, 0.20), 0.41 R^2^ = 0.04, 0.03	–0.02 (–0.15, 0.11), 0.78 R^2^ = 0.01, 0.53	–0.01 (–0.12, 0.10), 0.79 R^2^ = 0.03, 0.46
**Sodium-to-potassium ratio**				
Model 1	–0.01 (–0.09, 0.06), 0.70 R^2^ = 0.0002, 0.70	–0.02 (–0.09, 0.04), 0.47 R^2^ = 0.0008, 0.47	–0.03 (–0.10, 0.03), 0.33 R^2^ = 0.002, 0.33	0.01 (–0.06, 0.08), 0.82 R^2^ = 0.0001, 0.82
Model 2	–0.01 (–0.10, 0.07), 0.74 R^2^ = 0.01, 0.46	–0.02 (–0.09, 0.05) R^2^ = 0.03, 0.11	–0.04 (–0.11, 0.03), 0.28 R^2^ = 0.01, 0.62	0.01 (–0.06, 0.08), 0.83 R^2^ = 0.02, 0.16
Model 4^a^	-0.04 (-0.14, 0.06), 0.43 R^2^ = 0.04, 0.40	0.11 (-0.07, 0.09), 0.77 R^2^ = 0.04, 0.02	-0.05 (-0.12, 0.01), 0.11 R^2^ = 0.02, 0.33	0.01 (-0.05, 0.08), 0.69 R^2^ = 0.03, 0.40

Model 1: unadjusted

Model 2: age, socioeconomic disadvantage, weight category, day of urine collection

Model 3: model 2 + potassium where sodium is independent variable + sodium where potassium is independent variable

Model 4: Model 2 + birth weight (kg)

Sex × urinary sodium interaction: *P* value = 0.15 (SBP z-score), *P* value = 0.42 (DBP z-score) (Model 2)

Sex × urinary potassium interaction: *P* value = 0.64 (SBP z-score), *P* value = 0.91 (DBP z-score) (Model 2)

Bold values indicate statistical significance *P* value < 0.05

Sex × urinary sodium-to-potassium ratio: *P* value = 0.93 (SBP z-score), *P* value = 0.40 (DBP z-score) (Model 2)

*SBP* systolic blood pressure, *DBP* diastolic blood pressure

^a^*n* = 605 due to missing data for birth weight *n* = 149 and *n* = 1 excluded as reported birth weight deemed implausible (297 g). Final sample size girls *n* = 239 and boys *n* = 336

### 24-hour urinary electrolyte excretion and blood pressure by weight category

**Fig. 2:**
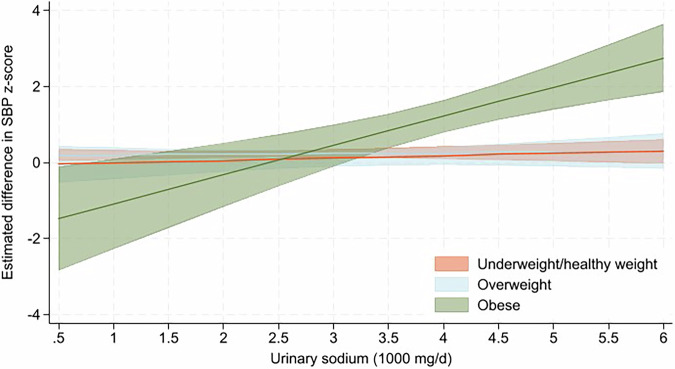
Modelled association between urinary sodium (1000 mg/d) and SBP z-score across weight categories ^1, 2, 3^. ^1^ Children grouped across three weight categories (underweight/healthy weight, overweight or obese). ^2^ Adjusted for age, sex, socioeconomic disadvantage, day of urine collection. ^3^ The straight line represents the modelled estimated difference in SBP z-score and the 95% confidence intervals are represented in shaded colours. Abbreviations: SBP systolic blood pressure

There was evidence of a significant interaction between sodium excretion and weight category on SBP z-score (urinary sodium × weight categories, grouped as underweight/healthy weight, overweight, and obese *p*value for interaction = 0.002). Results stratified by weight category showed a positive association between sodium excretion (1000 mg/d) and SBP z-score among children living with obesity. The association approached statistical significance (b-coefficient 0.75 [95% CI 0.00, 1.51], *p*value = 0.05; adjusted for age, sex, socioeconomic disadvantage, day of urine collection, *n* = 21,) (Fig. 2). The association was attenuated after additional adjustment for potassium excretion (b-coefficient 0.70 [95% CI –0.04, 1.43], *p*value = 0.06, *n* = 21) and in the reduced sample size where birth weight was included as a covariate (0.75 [95% CI –0.40, 1.89], *p*value = 0.18, *n* = 17) (Supplementary Table 1). There was no association between sodium excretion and SBP z-score among children who were underweight/healthy weight or overweight (Fig. 2). In sensitivity analyses, when children were grouped into two weight category groups (those who were underweight/healthy weight vs. overweight/obese) there was a non-significant positive association between 24-h urinary sodium excretion and SBP z-score among those with overweight/obesity (b-coefficient 0.11 [95% CI –0.03, 0.24], *p*value = 0.11, *n* = 112, adjusted for age, sex, socioeconomic disadvantage and day of urine collection) (Supplementary Table 1).

## Discussion

This cross-sectional analysis of Victorian schoolchildren aged 4–12 years found a mean daily sodium excretion of 2419 mg (6.2 g salt) and that approximately three quarters of children exceeded the recommended upper level of sodium intake for Australian children [34]. Whilst in the unadjusted regression model there was a positive association between urinary sodium excretion and SBP z-score (1000 mg/d increase in sodium associated with ~0.08 mmHg higher SBP), this association was attenuated and no longer statistically significant after adjustment for covariates. However, a positive association was found in children among girls and children living with obesity. Consistent with other paediatric studies, no associations between urinary electrolyte excretion and DBP were found [2, 6, 9, 13].

Although there is evidence to suggest sodium intake does positively affect SBP from a young age [9], some studies have also observed null findings regarding this association [13, 15, 22, 36, 37]. In their systematic review, Leyvraz et al. (2018) noted that the impact of sodium intake (measured via urinary and dietary methods) upon SBP appeared stronger among infants (< 1 years of age) and children >12 years of age [9]. In our study, sodium intake (2419 mg/d) is lower than that seen in other paediatric samples globally (mean sodium intakes 3000 mg/d to 3387 mg/d), where significant associations of sodium excretion or intake with SBP have been observed [7, 9, 38]. Only one other Australian study examined the association between sodium intake and blood pressure in children aged 9-years with a similar sodium intake (boys: 2640 mg/d; girls 2400 mg/d) and found no significant association with blood pressure in boys or girls [37]. However, this study utilised a food frequency questionnaire, which is unable to accurately measure sodium intake [20].

Although the interaction between sex and urinary sodium on SBP did not reach statistical significance, sex-stratified analyses revealed a positive association in girls but not boys. Among girls a 1000 mg/d increment in sodium was associated with a 0.10 higher SBP z-score (equivalent to ~1.0 mm Hg SBP). Similar effect sizes (1000 mg/d increase sodium increasing SBP by ~1 mm Hg) have been found in a national sample of US children using multiple 24-h dietary recalls to measure sodium intake [7] and a meta-analysis of studies restricted to 24-h urinary sodium excretion [9]. While Yang et al. (2012) did not examine potential differences in the association by sex, Leyvraz et al.’s (2018) meta-analysis did report positive associations between sodium intake (measured by urinary or dietary methods) and SBP in both boys and girls. There is some limited evidence of a sex difference in the response to a reduction in sodium intake on SBP in children. In a 3-year randomised controlled trial of sodium reduction in adolescents, Sinaiko et al. (1993) reported a reduced rise in the slope of blood pressure among girls who were randomised to the low sodium diet over time, but not in boys; however, boys were also less compliant with the reduced sodium diet [39]. Campanozzi et al. reported a non-significant trend towards a positive association between 24-h sodium excretion and SBP in girls (*p* = 0.05), but not in boys, in their cross-sectional analyses of a national sample of Italian children aged 6–18 years [21]. It is unclear why sodium may have different effects on SBP between boys and girls. The exploratory findings related to sex differences reported in this study should be interpreted with caution and warrant further investigation in larger samples.

Body weight was found to significantly moderate the association between sodium excretion and SBP. In stratified analyses, a positive association between sodium excretion and SBP z-score was observed among children living with obesity. Although the confidence interval included zero, the magnitude of the association suggests a potentially clinically meaningful effect, with a 1000 mg/day increment in sodium excretion associated with a 0.75 higher SBP z-score, equivalent to ~7 mm Hg higher SBP. This positive association aligns with findings from previous studies [9], although we acknowledge the large uncertainty around this estimate, as indicated by the wide 95% confidence interval and the small number of children with obesity (*n* = 21) in the present study. In a seminal study of 11–14 year old American schoolchildren, which included 7 repeated 24-h urine collections, Cooper et al. (1980) found that children who were ≥109lbs (49 kg) exhibited a significant association between 24-h sodium excretion and SBP—whereas their lower-weight peers did not [38]. Among a national sample of US children aged 8-18 years, Yang et al. (2012) found that a 1000 mg/day increase in dietary sodium intake resulted in a 6% increase in the risk for pre-high blood pressure/high blood pressure in normal weight children and adolescents, compared to a 74% increase in risk among overweight/obese participants [7]. This is consistent with a recent systematic review which found that the association between sodium intake and SBP was stronger among children with overweight/obesity [9]. A mechanism which might explain this association is that salt sensitivity—the degree to which sodium intake raises blood pressure—may to some extent be influenced by excess body weight [10].

We found a positive association of potassium excretion and SBP in the overall sample in the unadjusted model only. This may be because potassium is widely distributed across food items and may reflect total energy intake rather than a high intake of fruits and vegetables, as a mean potassium excretion of 1813 mg/d indicates a lower than recommended intake of dietary potassium [34]. Studies assessing the effect of dietary or urinary potassium on blood pressure in children are sparse, and more conflicting than for sodium [40]. Two studies in children, using urinary potassium excretion found no association of potassium or sodium-to-potassium ratio with BP, consistent with our findings [22, 41]. In contrast, Geleijnse et al. (1990) found in a Dutch cohort of children aged 5–17 years that 24-h urinary potassium, but not sodium (estimated from repeated annual overnight urine samples), was associated with a reduction in the mean age-related rise in SBP. They also observed a positive association between the sodium-to-potassium ratio and change in SBP, with a stronger effect among children aged 14–17 years [15]. Studies using dietary recall methods have indicated higher potassium intake with lower blood pressure and higher sodium-to-potassium ratios with higher BP [13, 15, 42]. In a large national sample of US adolescents aged 12–14 years using 24-h diet recall, the odds for high SBP were significantly lower among those with higher potassium intake (≥ 2800 mg/d) and significantly higher for those with a higher sodium-to-potassium ratio (≥ 2.5) [42]. Longitudinal studies using dietary data have also shown an inverse relationship between potassium intake and SBP [13, 15]. A 2013 meta-analysis assessed the impact of randomised, controlled interventions to increase dietary potassium intake (two used potassium supplementation) on blood pressure in childhood [2]. This analysis found no effect of higher potassium intake on BP [2]. In summary, the potential influence of potassium on blood pressure may be more likely during later childhood and adolescence and may take time to manifest.

Strengths of this study include the use of 24-h urinary sodium excretion as an objective marker of sodium intake within a large sample of children. This method has been shown to capture ~93% of daily dietary sodium consumed [20, 43]. Other strengths of this study include the use of standardised protocols to measure blood pressure and the use of BP z-scores [33] which account for differences in blood pressure by sex, age, and height. There are also several limitations present in this study. First, the convenience sampling method used introduces potential bias in participants volunteering to take part. This combined with the low response rate of 7% limits the generalisability of these findings to the broader paediatric population. Second, the use of 24-h urine collection is not as accurate for measurement of potassium as it is for sodium—this is likely to have resulted in a lower estimate of daily potassium intake [44]. Third, a single 24-h urine sample does not reflect habitual intake of sodium and potassium and the large intra-individual variation associated with urinary electrolyte excretion can lead to biased estimates when assessing diet and health associations [45]. Fourth, data on physical activity, which is known to influence blood pressure levels [46] was not collected and could not be adjusted for in the analysis. Finally, this was a cross-sectional study and there may be residual confounding.

In conclusion, this was the first study in an Australian paediatric population to use 24-h urinary sodium to assess the association between sodium intake and blood pressure. Sodium intake in this sample exceeded recommended levels for healthy development. Although no overall association was found between urinary sodium excretion and BP in the regression models adjusted for covariates, we did find a positive association between 24-h urinary excretion and SBP among girls and in children living with obesity, providing further support to the hypothesis that body weight is a moderator of this relationship through heightened salt sensitivity. Longitudinal studies utilising repeated 24-h urine collection are required to further understand the effect of sodium and potassium on paediatric blood pressure levels, particularly in relation to the influence of excess body weight and potential sex differences. A higher BMI throughout life from childhood is known to be an independent predictor of elevated BP [47]. Therefore, public health interventions aiming to reduce the elevated cardiovascular risk of raised BP during childhood are likely to be most effective by focussing on reducing sodium intake in conjunction with promoting healthy weight.

## Supplementary information

Supplementary information
