# Supplementary material for: Sodium, potassium and blood pressure in Australian schoolchildren: exploring differences by sex and weight status—a cross-sectional study
Source: Hypertens Res. 2026 Jan 18;49(3):938–48. doi: 10.1038/s41440-025-02489-1 (PMC12960245; doi:10.1038/s41440-025-02489-1)
Supplement: Supplementary file 1 — Supplementary information [file 41440_2025_2489_MOESM1_ESM.docx]

**Supplementary Table 1.** Association between 24-hour urinary sodium (1000 mg/d) with blood pressure z-scores among children aged 4-12 years participating in the Salt and Other Nutrient Intakes in Children study, stratified by weight category

| **Blood pressure** | **Model 1** | **Model 2** | **Model 3** | **Model 4^1^** |
| --- | --- | --- | --- | --- |
|  | **b-coefficient (95% CI), p-value**  **R^2^, *P* value** | **b-coefficient (95% CI), p-value**  **R^2^, *P* value** | **b-coefficient (95% CI), p-value**  **R^2^, *P* value** | **b-coefficient (95% CI), p-value**  **R^2^, *P* value** |
| **SBP z-score** |  |  |  |  |
| Underweight/healthy weight (n=643) | 0.06 (-0.02, 0.13), 0.13  R^2^=0.003, 0.13 | 0.03 (-0.05, 0.11), 0.49  R^2^=0.02, 0.27 | 0.02 (-0.06, 0.10), 0.63  R^2^=0.02, 0.35 | 0.04 (-0.06, 0.14), 0.39  R^2^=0.03, 0.02 |
| Overweight (n=91) | 0.09 (-0.06, 0.23), 0.24  R^2^=0.01, 0.24 | 0.04 (-0.13, 0.210), 0.65  R^2^=0.10, 0.13 | 0.03 (-0.15, 0.20), 0.76  R^2^=0.10, 0.22 | 0.05 (-0.16, 0.27), 0.60  R^2^=0.11, 0.13 |
| Obese (n=21) | **0.78 (0.36, 1.22), 0.001**  R^2^=0.30, 0.001 | 0.75 (0.00, 1.51), 0.05  R^2^=0.53, <0.001 | 0.70 (-0.04, 1.43), 0.06  R^2^=0.59, <0.001 | 0.75 (-0.40, 1.89), 0.18  R^2^=0.59, <0.001 |
| **SBP z-score** |  |  |  |  |
| Underweight/healthy weight (n=643) | 0.06 (-0.02, 0.13), 0.13  R^2^=0.004, 0.13 | 0.03 (-0.05, 0.11), 0.49  R^2^=0.02, 0.27 | 0.02 (-0.06, 0.10), 0.64  R^2^=0.02, 0.35 | 0.04 (-0.06, 0.14), 0.39  R^2^=0.03, 0.02 |
| Overweight/Obese (n=112) | **0.17 (0.01, 0.28), 0.004**  R^2^=0.04, 0.005 | 0.11 (-0.03, 0.24), 0.12  R^2^=0.11, 0.002 | 0.08 (-0.06, 0.22), 0.25  R^2^=0.12, 0.004 | 0.08 (-0.10, 0.27), 0.37  R^2^=0.11, 0.03 |
| **DBP z-score** |  |  |  |  |
| Underweight/healthy weight (n=643) | -0.02 (-0.09, 0.06), 0.64  R^2^=0.02, <0.001 | -0.03 (-0.11, 0.04), 0.41  R^2^=0.02, 0.002 | -0.02 (-0.09, 0.06), 0.64  R^2^=0.02, <0.001 | -0.02 (-0.11, 0.07), 0.64  R^2^=0.14, 0.02 |
| Overweight (n=91) | -0.02 (-0.15, 0.11), 0.79  R^2^=0.11, 0.01 | -0.03 (-0.15, 0.07), 0.50  R^2^=0.10, 0.008 | -0.02 (-0.15, 0.11), 0.79  R^2^=0.11, 0.01 | -0.02 (-0.11, 0.07), 0.64  R^2^=0.14, 0.03 |
| Obese (n=21) | 0.04 (-0.38, 0.46), 0.84  R^2^=0.002, 0.84 | 0.37 (-0.28, 1.02), 0.24  R^2^=0.21, 0.03 | 0.34 (-0.27, 0.93), 0.25  R^2^=0.30, 0.04 | 0.33 (-0657, 1.31), 0.48  R^2^=0.30, <0.001 |
| **DBP z-score** |  |  |  |  |
| Underweight/healthy weight (n=643) | -0.04 (-0.11, 0.03), 0.22  R^2^=0.002, 0.22 | -0.03 (-0.10, 0.04), 0.41  R^2^=0.02, 0.002 | -0.02 (-0.09, 0.06), 0.64  R^2^=0.02, <0.001 | -0.02 (-0.09, 0.05), 0.64  R^2^=0.04, <0.001 |
| Overweight/Obese (n=112) | 0.003 (-0.08, 0.09), 0.27  R^2^=0.000, 0.94 | -0.006 (-0.10, 0.09), 0.89  R^2^=0.08, 0.007 | 0.001 (-0.10, 0.11), 0.98  R^2^=0.08, 0.006 | 0.004 (-0.09, 0.09), 0939  R^2^=0.10, 0.02 |

Model 1: unadjusted

Model 2: age, sex, socioeconomic disadvantage, day of urine collection

Model 3: model 2 + potassium intake

Model 4: Model 2 + birth weight (kg).

**^1^** Reduced sample size due to missing data for birth weight n=149 and n=1 excluded as reported birth weight deemed implausible (297g). Final sample size: Underweight/healthy weight n=512; Overweight n=76; Obese n=17. Underweight/healthy weight n=512; Overweight/obese n=93

Bold values indicate statistical significance *P* value <0.05

Abbreviations: SBP systolic blood pressure; DBP diastolic blood pressure
